# Challenges of Care Transition From Hospital to Home for Older Colorectal Surgery Patients: Surgeons’ Perspectives

**DOI:** 10.1097/AS9.0000000000000636

**Published:** 2025-12-29

**Authors:** Sevdenur Cizginer, Ferhat Yildiz, Christy E. Cauley, Stephen J. Bartels, Christine S. Ritchie, Bharati D. Kochar, Matthew D. Vrees, Richard N. Jones, Edward R. Marcantonio, Rocco Ricciardi, Benjamin F. Crabtree

**Affiliations:** From the * Center for Aging and Serious Illness, Mongan Institute, Massachusetts General Hospital, Boston, MA; † Division of Palliative Care and Geriatric Medicine, Department of Medicine, Massachusetts General Hospital, Boston, MA; ‡ Harvard Medical School, Boston, MA; § Mongan Institute, Massachusetts General Hospital, Boston, MA; ‖ Department of Surgery, Massachusetts General Hospital, Boston, MA; ¶ Division of Gastroenterology, Department of Medicine, Massachusetts General Hospital, Boston, MA; # Department of Psychiatry and Human Behavior, Warren Alpert Medical School of Brown University, Providence, RI; ** Divisions of General Medicine and Gerontology, Department of Medicine, Beth Israel Deaconess Medical Center, Boston, MA; †† Department of Surgery, The Miriam Hospital, Providence, RI; ‡‡ Warren Alpert Medical School of Brown University, Providence, RI; §§ Division of Research, Department of Family Medicine and Community Health, Rutgers Robert Wood Johnson Medical School, New Brunswick, NJ; ‖‖ Cancer Prevention and Control Research Program, Rutgers Cancer Institute of New Jersey, New Brunswick, NJ.

**Keywords:** Colorectal surgery, care transition, geriatric surgery, older surgical patients, readmission, postdischarge outcomes, qualitative methods

## Abstract

**Objective:**

Postdischarge transitions from the hospital to home in older (≥65 years) colorectal surgery patients have a high risk of medication errors, complications, and worsening of existing conditions. Up to 14% are readmitted within 30 days, costing ~$180 million annually. The anticipated 50% increase in colorectal cancer surgeries in older adults by 2040 necessitates an improvement in care transitions and outcomes.

**Methods:**

We conducted semi-structured qualitative interviews with 10 surgeons from 8 US health systems to inform the design of a multicomponent care transition model. We selected participants through stratified purposive sampling based on experience with older surgical patients and/or leadership roles. Consolidated Criteria for Reporting Qualitative Studies guidelines were followed, and a detailed line-by-line editing and organizing style was used to analyze transcripts.

**Results:**

The interviews identified challenges in care transitions and potential solutions, and 4 themes emerged: (1) Discharge planning should start before surgery and incorporate preoperative geriatrics evaluation and planning; (2) Coordinated communication and collaboration among multidisciplinary care teams are necessary but often lacking; (3) Educating older surgical patients and their care partners and involving them in care decisions is needed for successful management of care responsibilities after discharge; and (4) The complex and fragmented healthcare system creates care challenges postdischarge.

**Conclusions:**

Discharge planning that begins preoperatively, integrates geriatrics domains, ensures timely and coordinated interdisciplinary communication postdischarge, and emphasizes patient and family education is essential to improve care transitions in older colorectal surgery patients. A multilevel care transition model incorporating these elements may enhance outcomes and reduce readmissions.

## INTRODUCTION

Care transitions for older adults (≥65 years old) after colorectal surgery pose significant challenges. There is an increased risk of postdischarge complications, including medication errors, exacerbation of existing conditions, and new health issues. Nearly 14% experience readmission within 30 days, costing an estimated $180 million annually in the United States.^1–9^ With the number of colorectal cancer surgeries in older adults projected to increase by 50% by 2040,^10^ there is a need to enhance postdischarge care and reduce costly readmissions.

Despite the risks of care transitions, no optimum care transition model improves postdischarge outcomes.^11^ Ostomy creation, change in fluid balance, oral intake, and bowel function complicate the postdischarge period, especially in patients with multiple comorbidities and functional or cognitive impairment. Moreover, the complexity and fragmentation of the healthcare system in the United States often hinder seamless transitions from hospital to home, contributing to adverse outcomes.^12,13^ Perioperative planning,^14–16^ interdisciplinary communication,^17–19^ and patient-centered education^20–22^ are key components of effective care transitions for this population. However, these components are often separately or inconsistently implemented without a clear patient benefit. To address these issues, we need a tailored, evidence-based intervention.

Here, we conducted semi-structured qualitative interviews with surgeons from diverse health systems to identify challenges in care transitions for older colorectal surgery patients. Interview transcripts were rigorously and qualitatively analyzed, and key themes were identified. These insights will inform the development of a multicomponent care transition model to improve postdischarge outcomes in this patient group.

## METHODS AND SETTINGS

We included 8 US health systems, each with a dedicated colorectal surgery team. The systems represented a broad spectrum of healthcare settings, including the Veterans Affairs healthcare system, urban, rural, and academic medical centers, community healthcare systems, and tertiary care institutions. These systems were selected based on (1) having an active surgery team that performs major elective colorectal surgeries, (2) representing different healthcare system types (open and closed, Veterans Affairs) and care settings (urban, rural, and academic) to capture various patient populations, (3) involving surgeons who manage high volumes of colorectal surgery patients and maintain heavy clinical workloads, (4) involving surgeons with a special interest and expertise in geriatric surgery, and (4) involving surgeons with administrative roles.

### Design and Data Collection

We conducted a qualitative study using semi-structured interviews with surgeons with expertise in geriatric surgery, adhering to the Standards for Reporting Qualitative Research.^23^ The study was exempt from the Mass General Brigham Institutional Review Board. The principal investigator (S.C.) and the fellow (F.Y.) recruited participants and conducted interviews between September 2023 and April 2024. Participants were identified using stratified purposive sampling and recruited through email solicitation and snowball sampling. The “informative power” principle informed the sampling strategy and guided sample size determination.^24^ We iteratively and purposely recruited participants until we reached thematic saturation consistent with qualitative research methods.^24,25^ Participants were compensated with a $75 gift card.

### Interview Format and Process

We interviewed participants using an institutionally approved secure online video platform. We used an interview guide with open-ended questions and follow-up probes. Interview questions gathered perspectives on (1) general issues with the transition of care after discharge, (2) ideal care transition team members that should be part of the care after discharge, and (3) ideal outcomes for an older patient 1 month after colorectal surgery (see Table 1). Follow-up probes were used to ensure we obtained information on specific issues (eg, nutrition, fluids, medications, ideal collaboration among team members, and strategies to reach an ideal care transition).

**TABLE 1.** Interview Grand Tour Questions

Case Example	In order to get your opinions about transition of care in older colorectal surgery patients, I would like to get your reactions to a case example: Mr. Thomas is a 78-year-old patient with underlying health conditions including diabetes, chronic heart failure, chronic kidney failure, atrial fibrillation, and depression. He was recently diagnosed with colon cancer and underwent a colectomy. Mr. Thomas is now back at home and hoping for a smooth recovery without the challenges of complications or need for returning to the hospital.
1	What types of issues would you be considering when planning to discharge a patient like Mr. Thomas with multiple health conditions, on multiple medications, returning home after a colectomy for colon cancer?
2	In an ideal scenario, could you tell me who should be involved in the postdischarge care for older patients who have had colorectal cancer surgery?
3	Could you tell me about the best possible outcomes for an older patient one month after discharge following a colon cancer surgery?

### Data Analysis and Interpretation

Iterative reflexivity^26^ was conducted throughout data collection, analysis, and interpretation. After each interview, 3 authors (S.C., F.Y., and B.F.C.) held a reflexivity session, followed by 2 authors (S.C. and F.Y.) writing memos for each interview. Interview questions were revised when needed. The analysis followed an “editing-organizing style,”^27^ where investigators first read the transcripts line-by-line to identify units and segments relevant to their research, then expanded observations and comments, revised and sorted segments to make connections to support interpretation and theme identification.

Three authors (S.C., F.Y., and B.F.C.) read each interview, 2 (S.C. and F.Y.) analyzed 174 pages of transcript line-by-line, recording expanded observations, and all 3 (S.C., F.Y., and B.F.C.) had a detailed discussion to identify interview themes. An inductive, iterative process was used to reach a consensus, and then the themes were discussed with other coauthors.

Once interview themes were identified, the authors (F.Y. and S.C.) compared themes across interviews, identifying overarching themes. This was followed by in-depth discussions among the research team (F.Y., S.C., and B.F.C.) until a consensus was reached. In the final step, the interpretation process included an explanation by the research team (F.Y., S.C., and B.F.C.), which provided explanations for each theme, including interview summaries and related quotes.

## RESULTS

Each participant surgeon had a dedicated clinical focus on geriatric surgical care, and 7 also had administrative roles (see Table 2). Seven of 10 were female with more than 20 years of experience. One had less than 10 years of experience.

**TABLE 2.** Practice Descriptions and Characteristics of Surgeons Interviewed

Surgeon #	Setting Type	Specialty	Administrative Role	Years in Practice	Gender
1	Veterans Affairs	Colorectal Surgeon	Chief of Surgery	10–14	Female
2	Academic	Colorectal Surgeon	None	15–19	Female
3	Academic	Colorectal Surgeon	Medical Director	20–24	Female
4	Community	Colorectal Surgeon	None	0–4*	Female
5	Academic	Colorectal Surgeon	Division Director	20–24	Female
6	Academic	Bariatric Surgeon	Chief Medical Officer	25–29	Male
7	Veterans Affairs—Academic	Colorectal Surgeon	Chief of Surgical Services	20–24	Female
8	Community	Thoracic Surgeon	Chief of Surgery	40–44	Male
9	Community	Colorectal Surgeon	None	25–29	Male
10	Academic	Trauma Surgeon	Director, Geriatric Surgery	20–24	Female

* Five to 9 years of experience with geriatric surgery.

Each interview lasted at least 50 minutes, with the respondents reflecting on the challenges of care transition. Four themes (for themes and supporting quotations see Supplemental File 1, https://links.lww.com/AOSO/A557) resonated across all 10 interviews: (1) Preoperative planning and evaluation that incorporates a focus on geriatrics is necessary for successful recovery and postdischarge care; (2) Coordinated communication and collaboration among multidisciplinary teams involved in care are essential but often lacking; (3) Educating older surgical patients and their families, involving them in care decisions, and addressing social determinants are key to ensuring patients can manage their care responsibilities; and (4) The complex and fragmented healthcare system creates challenges for postdischarge care.

### Theme 1: Discharge Planning Should Start Before Surgery and Incorporate Preoperative Geriatrics Evaluation and Planning

Nearly all surgeons emphasized that preparation for postdischarge care must start well before the surgery to ensure a smooth transition and optimum patient outcomes. As one surgeon said,

“the key to this, and this is something we learned the hard way, is what you are going to decide for the discharge starts well in the preoperative period.”(S09)

Assessing patients’ nutritional, functional, and cognitive baseline status was identified as a key step, with relevant disciplines involved to address any deficiencies before surgery and plan for patients’ potential needs following discharge. As one surgeon said,

“That way (by determining baseline status), we can start to plan for what he’s going to need after discharge before we even operate on such a patient.” (S01)

Most participants emphasized the need for thorough documentation of those evaluations, and the patient’s baseline status and progress, beginning in the preoperative phase. Surgeons stressed the importance of recording baseline comorbid conditions, particularly heart and kidney failure status, and clear documentation of preoperative home medications and their indications. Additionally, they highlighted the need to assess baseline geriatrics domains—functional, cognitive, and nutritional—and social determinants of health. This record-keeping aids in understanding the patient’s clinical trajectory and enables comparisons between preoperative and postoperative conditions, facilitating the development of a tailored follow-up plan after discharge. Most participants highlighted the importance of assessing whether the patient would return home alone, have caregiving support in place, or if the home environment was suitable for recovery (eg, stairs to the bedroom). They noted that addressing these concerns after the surgery omitted the opportunity to make necessary adjustments. A typical response underscored the importance of assessment timing:

“So these really sort of simple things, these non-medical things that I think really can help anticipate sort of a soft landing when patients get home. Because once you’re sitting there and you’re the discharge planner and it’s the day of discharge, you can’t really make all those things happen” (S03)

### Theme 2: Coordinated Communication and Collaboration Among Multidisciplinary Teams Involved in Care are Necessary But Often Lacking

Although most surgeons articulated themselves as the primary responsible provider after discharge, they accepted that they have neither the bandwidth nor expertise to address each issue.

“The problem is for us anyway, is that there’s just so many moving parts to keep track of. It’s hard. And then we almost need a proxy to help take care of it because we’re not able to get to, you know, sometimes we’re in the operating room for hours and hours and hours. If there’s something major going on, we don’t even know for the whole day. So somebody else is triaging it.” (S02)

An interdisciplinary team approach is important during the postdischarge period, and while it often exists, participants identified a lack of timely and coordinated communication among disciplines. While many people such as physical therapists, nurses, surgical team members, and primary care physicians (PCPs) are involved during this period, defined roles, expectations, and routes of effective 2-way communication are missing. As stated in one of our interviews:

“So I’d say that’s definitely an Achilles heel, that there’s not… People are doing it in pockets, but there’s no systematic way, no harmony, no timely and effective communication between all moving pieces.”(S03)

As a remedy, surgeons suggested designating a point of contact, such as a registered nurse, who could serve as a central hub to coordinate timely communication among all parties. Several observed that communication is more straightforward within a closed health system, where all disciplines are integrated, compared with an open health system, where communication barriers can arise.

A robust, warm handoff between surgeons and PCPs was important to prevent care gaps. However, they all reluctantly admitted that a closed-loop communication system between them and other providers after the patient’s discharge did not exist:

“But I think that’s something that we increasingly sort of fall back and rely on the fact that we write a discharge summary, and it gets faxed to the referring doctor and we don’t really know, whether that actually got in front of that doctor or if that doctor has some ideas about it, how that would get back to us.”(S03)

### Theme 3: Educating Older Surgical Patients and Their Families and Involving Them in Care Decisions is Needed to Ensure Patients Can Manage Their Care Responsibilities After Discharge

Surgeons emphasized that educating patients and their families should begin before surgery and continue throughout the care. They indicated that ongoing education helps patients recognize potential problems early and take appropriate action. Some respondents noted that educating older adults may require repetition and the “repeat-back” method to ensure understanding. They also noted the important role of family involvement, especially as home-based treatments increase. For instance, educating family members in ostomy and wound care was identified as necessary to ensure families/carepartners were confident in providing care. Key strategies to foster a sense of ownership and understanding included engaging patients and their families in care decisions, understanding care goals, and explaining complications and outcomes. Respondents described the family as a coaching team and emphasized equipping them with the resources to support the patient’s recovery at home. Several expressed concern about who would be at home to check on and support the patient during the early period after discharge, an important time for recovery.

“So definitely we need to know a family i.e., caregiver of some sort is available to be with the patient for the first couple of weeks or so.” (S05)

As a case in point, a community colorectal cancer surgeon used a case example to emphasize the potential consequences of not addressing patient and family education before surgery:

“Maybe I saved her life by doing that surgery. But was this… I didn’t even stop to ask any questions. You know, I just. Yeah, she was here. I told her risk and benefits went ahead and did the surgery. Fortunately, you know, she made it. But one thing is, what is her life going to be now? She cannot manage her own colostomy. Nobody wants to come near her in her family to take care of her. I mean, nobody had even discussed any of these issues.” (S09)

He went on to emphasize the importance of assessing how well that education was understood by patients and their families, saying:

“It is for them to speak to tell us what you understood, you know, because a lot of what we try to explain to them just, you know, shoots past them.” (S09)

Educating patients and involving them in care decisions ensures they clearly understand why the procedure is necessary and what potential complications might arise. As one surgeon noted:

“So it’s quite possible they would say yes, but they’re not happy with the ostomy. But once they come to know that it was absolutely necessary, I think most of them are very understanding.” (S07)

Similarly, another surgeon noted that while family members want to help the patient, they sometimes lack the knowledge and resources to do so;

“They (family) want to help, and they don’t know how. And I think that we could be better at letting them know very specifically how they can help.” (S03)

Time constraints for surgeons were identified as a substantial barrier to effective communication with patients and families. A care transition coach nurse was suggested as a potential solution to provide education to patients and families from preoperative through the postdischarge period.

### Theme 4: The Complex and Fragmented Healthcare System Creates Care Challenges Postdischarge

One surgeon described patients leaving the hospital and stepping into “a black hole,” a sudden disconnect from the comprehensive care they received during their hospital stay. Although multiple disciplines should remain involved in postdischarge care, this was challenging for older patients to understand and navigate. Respondents felt the healthcare system posed additional difficulties, such as when a patient needs to visit a separate emergency room near their home that lacks information about their procedure and hospital stay. Interviewees often noted that navigating this complex system and ensuring follow-up care fell on the patients and their families. They expressed concerns about the transition patients experience upon discharge:

“I think sometimes it’s hard for us to reach people once they’ve gotten discharged. Like calling in and checking in on them. I think, they go from a setting where there are in the hospital where there are doctors, nurses, case managers, social workers, physical therapists, nutritionists, like so many people who are involved in their care, and then they go home. And we don’t really have any things set up for them to continue to interact with us unless there’s any issue because they can always call.” (S04)

A number of these surgeons noted that patients and their families feel overwhelmed by the number of people involved in their care and do not know who to contact when issues arise:

“The health care system, particularly for older adults, but really for anybody, it’s just too hard to navigate. It’s too complex. There are too many specialists. There’s, you know, too many phone numbers.” (S10)

Interviews showed surgeons view patients and their families as often burdened with managing their care logistics, as this surgeon further noted:

“… that’s where I think things get confusing because oftentimes, they’ll call the surgeon’s office and then they’ll get directions to call. Okay, so call CAT scan to make this appointment. And it puts the onus on the patient and the family to be the administrator.” (S10)

Another surgeon mentioned that patients struggled to directly connect with the appropriate healthcare provider because of the layers within the healthcare system:

“There’s not an easy way for patients to actually talk to somebody. So there’s tons of ways you can send a message or you can do this or that, but there’s no direct... It’s not easy for somebody to just pick up the phone and say, can I talk to whoever it is, whomever, and then actually get them.” (S02)

## DISCUSSION

Care transition after major colorectal surgery is complex, with significant short- and long-term risks for older patients.^1–4^ This study identified that preoperative planning that integrates geriatrics evaluation, patient/family/carepartner education, and clear communication pathways among hospital team members and across the broader healthcare system matrix that the patient navigates are essential to facilitate complication-free recovery and reduce readmission. While evidence-based models—such as Care Transition Intervention^28^ and structured interdisciplinary models that integrate geriatrics principles (eg, surgery comanagement programs^29^)—propose solutions to address those major elements, the development and implementation of such a comprehensive care transition model has not been rigorously tested in surgical patients.

The necessity for preoperative start for discharge planning, rather than being confined to the inpatient period, emerged as a central theme. Current practices focus on discharge processes during hospitalization, such as postoperative assessments, documentation, and handoff communication at the time of discharge.^30,31^ However, surgeons argue that this approach results in a poor understanding of the patient’s baseline health, hampers the establishment of realistic care goals, and limits the ability to effectively monitor patient progress after discharge. Additionally, critical preparations—such as home environment adjustments, food stocking, and arranging support systems—are frequently overlooked, leaving patients and families inadequately prepared to manage recovery after major abdominal surgery.

Surgeons consistently highlighted the importance of preoperative assessments of baseline comorbidities, particularly severity of heart failure, renal failure, and geriatrics domains such as nutrition, cognition, functional status, home medications, and social determinants of health. They noted that while cardiac and anesthesia risk assessments are typically robust, geriatrics evaluations remain insufficient. Patients with poor preoperative physical function or inadequate home support require additional information and resources because they are at high risk for poor coping capacity, complications, and readmission after discharge.^32,33^ Additionally, postsurgical challenges such as unpredictable bowel function after surgery or ostomy creation necessitate adjustment strategies,^34^ a process further complicated by cognitive or functional impairments. Detailed information on geriatrics domains can guide PCPs, surgeons, oncologists, nutritionists, and physical/occupational therapists to create informed, tailored plans to monitor patient progress and manage high-risk medications following discharge. While integrating geriatrics into preoperative evaluation is necessary, surgeons emphasized that geriatrics assessment and planning fall outside their expertise and are not feasible within their time constraints.

Indeed, surgery programs that incorporate geriatrics experts, such as geriatricians, achieve better outcomes for older surgical patients.^29,35–38^ These models can inform the development of care transition models that integrate geriatrics expertise. Technology-driven programs, such as mobile applications,^39^ and integrated care models, including nurse-led transition programs, have improved patients’ perceptions of continuity of care.^40^ In contrast, other interventions (eg, structured telephone follow-ups, home visit programs, and case manager led interventions) have not reduced readmission rates and show limited impact on quality of life or emergency visits.^41–43^ Notably, none of those interventions begin in the preoperative phase or include geriatrics domains, 2 major themes identified in our interviews.

The transition home postsurgery is perilous, as patients lose the systematic support of the hospital and face fragmented care. The “black hole” has communication breakdowns and no central point of contact. The recovery burden is transferred from the clinical team to the patient or care partner. Surgeons stressed that patient and family education could help but should start preoperatively and continue throughout the care journey. They suggested the “repeat-back” method to ensure understanding. Educational efforts empower patients and families to manage care effectively, reduce complications, anxiety,^44^ and align with the broader literature on patient-centered care.^45,46^ Establishing a central “care transition coach” could streamline communication and guide patients.

Our study has several strengths that enhance its impact and relevance. First, it provides a unique perspective by capturing insights directly from surgeons, mostly from colorectal surgery, but also other disciplines. All had expertise in geriatric surgery, which is often underrepresented in care transition discussions. This focus allowed us to identify gaps in care transition planning with practical, clinician-informed recommendations for older surgical patients. Second, our study emphasized the role of often overlooked baseline geriatrics assessments in discharge planning and highlighted specific geriatrics domains that influence patient outcomes. Third, our findings suggest actionable strategies such as integrating geriatrics experts into the process, using a transition coach as a point person for streamlining communication, and proactive patient and family education preoperatively. Finally, the study’s applicability is strengthened by its emphasis on interdisciplinary coordinated collaboration and alignment with emerging care models that integrate geriatrics and surgery principles to improve outcomes for vulnerable older adults.^29,38^ This qualitative analysis is subject to limitations. Though sufficient for thematic saturation, the sample size may not represent all settings (eg, rural-urban, racially, and economically diverse). Additionally, the perspectives gathered from surgeons with geriatrics expertise might differ from those without geriatrics experience. These factors limit the generalizability of the findings; however, qualitative investigation is not designed for generalizability. Future studies should include more diverse settings and actively include patients and care partners to codevelop strategies that reflect their unique needs and preferences. This process should also engage a diverse group of care providers, including geriatricians, advanced care practitioners, PCPs, nurses, social workers, physical therapists, and nutritionists, to ensure a more holistic approach to care transitions in older surgical patients.

Our findings underscore the necessity of a multicomponent, geriatrics-informed care transition model for older colorectal surgery patients, beginning preoperatively and integrating geriatric domains, interdisciplinary communication, and patient-centered education. To address systemic gaps, we propose merging core elements of evidence-based models like the Care Transition Intervention^28^ with structured interdisciplinary collaboration models that integrate geriatrics principles like geriatrics surgery comanagement programs.^29^ Such a model would uniquely target the physical, cognitive, and functional vulnerabilities of this population while empowering care teams, patients, and families to navigate postdischarge challenges. By tailoring this approach through implementation science, we can standardize adaptable, evidence-driven transitions that reduce readmissions, and improve outcomes in this high-risk surgical cohort.

## Supplementary Material
