# Supplementary material for: Challenges of Care Transition From Hospital to Home for Older Colorectal Surgery Patients: Surgeons’ Perspectives
Source: Ann Surg Open. 2025 Dec 29;7(1):e636. doi: 10.1097/AS9.0000000000000636 (PMC13016182; doi:10.1097/AS9.0000000000000636)
Supplement: Supplementary file 1 [file as9-7-e636-s001.pdf]

Supplemental File 1. Themes and Supporting Quotations

Theme 1: **Discharge planning should start before surgery and incorporate preoperative geriatrics evaluation and planning.**	*I think the more you upload up front, it is important. So having a good clear understanding of all the potential issues that patient could get into. So knowing family situations, psychosocial issues, alcohol use, smoking use, knowing their comorbidities, knowing who's following those comorbidities… (S05)*<br><br>*I would start preoperatively and try and figure out what his functional status was preoperatively. And that way we can start to plan for what he's going to need postoperatively before we even operate on such a patient. (S01)*<br><br>*I think, you know, what I try to do in my practice is just to back it up a little bit. And if you identify someone who, when they're in your clinic for an elective operation, that's going to be high risk, try to do a little bit of that work upfront before they even make it to the hospital. (S03)*<br><br>*A lot of our discharge planning starts preoperatively and so I would really like to know what was done in the preoperative period to prepare him as well as his family for discharge. So does he live alone? Does he have support at home? This is going to be very important and we usually find out about that preoperatively. (S07)*
Theme 2: **Coordinated communication and collaboration among multidisciplinary teams involved in care are important but often lacking**	*Well, I've never had the ideal, which would be the that getting everybody together at the time of discharge and saying, this is the situation, this is what's going on. This is where he is right now. He's going to be discharged. (S06)*<br><br>*I mean I think having open, you know, open lines of communication between all of the groups. I think it can become hard sometimes when there are so many people involved. (S04)*<br><br>*The ideal would be multidisciplinary care because the primary care doctor is not going to be the expert in the potential surgical problems and the surgeon is not going to be the expert in the medical problems. So optimally, you need both. (S08)*<br><br>*I think having open, you know, open lines of communication between all of the groups. I think it can become hard sometimes when there are so many people involved. But I think for at the very least, the attending surgeon and the geriatrics attending having each other's cell phone numbers so that or at least like, emailing each other back and forth just to make sure that there are open lines of communication just because things can change and*

I think it'd be… Making sure that all parties feel comfortable reaching out to each other. (S04)

So I think the ideal and I think it also depends on how involved your PCP is. But, you know, with regards to wanting to help the patient make decisions, and I don't always find that 100%, which is sad that's how it should be. But ideally, we would love to have the patient discuss this with their PCP prior to moving forward with any type of therapy to make sure that they feel like it's appropriate. (S05)

Theme 3:
Educating older surgical patients and their families and involving them in care decisions is needed to ensure patients can manage their care responsibilities following discharge.

I serve a 100% veteran patient population, and a lot of them are not willing to admit when they have a problem. So they will sit at home until things are about as bad as they can be before they decide to come back. And so knowing someone else is there, watching them is very important. (S01)

I think a lot of patients when they come in, they're just so freaked out with a new diagnosis of cancer. And just hearing the nuts and bolts of the surgery, which is really scary, that often they don't think about the other side of the hospitalization. So spending a little time to set expectations about what their potential function or functional limitations are going to be so that they have that window of time between when they saw the surgeon and when they come in for the surgery or they can arrange for family member or friends or family to be there when they get home. (S03)

The patient should always be involved as much as possible. And optimally, the patient should be the primary observer of his or her state. You know, of course, we know that in older adults that's not always the case. (S08)

So, I mean they just need to know about all the different ways of dealing with this cancer upfront and what their goals are obviously about their long term... I know what they expect and what they hope to be like and what would it be like if they couldn't be returned to their normal function, how they would feel about that. So I think as much as I can upload upfront. It helps me sort and move the patient through their process as easily as possible. (S05)

I think ideally it would be nice for them to understand how surgery and changes, whether it's their metabolism or their you know, it changes their steady state. And that ideally they would understand and they would be willing to interact with their primary care physicians to have those conversations. (S01)

Theme 4:

**The complex and fragmented health care system creates care challenges post discharge.**	*That's hard, right? That's an access problem. That's. That's the hardest thing to figure out and how to get them in to see their primary. Right. Have them with a set up appointment and then they need the education. (S01)*<br><br>*I mean, I think sometimes it's hard for us to reach people once they've gotten discharged. Like calling in and checking in on them. I think, they go from a setting where there are in the hospital where there are doctors, nurses, case managers, social workers, physical therapists, nutritionists, like so many people who are involved in their care, and then they go home. And we don't really have any things set up for them to continue to interact with us unless there's any issue because they can always call. (S04)*<br><br>*It's just too complicated. And I can talk about navigators and this, that and everything, but the health care system, particularly for older adults, but really for anybody, it's just too hard to navigate. It's too complex. There are too many specialists. There's, you know, too many phone numbers. (S10)*<br><br>*And then the family members, I think, we weigh heavily on them for those frail patients that the family member is going to take on a lot of that organization, post-op management, wound care. (S02)*<br><br>*People kind of go into a black hole when they get home. We do like maybe a little coaching about what they can eat, but there's really no one monitoring whether there's, you know, people are eating enough protein to recover from surgery. (S03)*
